# The Unexpected Diagnosis of Hepatic Tuberculosis in an Immunocompetent Patient

**DOI:** 10.1155/2020/7915084

**Published:** 2020-10-06

**Authors:** Nikolaos Garmpis, Christos Damaskos, Anna Garmpi, Aliki Liakea, Dimitrios Mantas

**Affiliations:** ^1^Second Department of Propedeutic Surgery, Laiko General Hospital, Medical School, National and Kapodistrian University of Athens, Athens, Greece; ^2^First Propedeutic Internal Medicine Department, Laiko General Hospital, Medical School, National and Kapodistrian University of Athens, Athens, Greece; ^3^First Department of Pathology, Medical School, National and Kapodistrian University of Athens, Athens, Greece

## Abstract

**Background/Aim:**

Tuberculosis (TB) is a chronic infectious disease which affects millions. The most affected system is the respiratory. Thus, hepatic TB (HTB) without involvement of other organs is not common. Its clinical manifestations are not specific, and both imaging and histopathological findings are necessary for the diagnosis. The differential diagnosis includes primary and metastatic liver malignancies. Our aim is to describe the rare entity of HTB via a case presentation. *Patient and Methods*. We report a case of a 50-year-old female with abdominal pain, weight loss, fever, and anorexia. All imaging methods described a liver lesion. She underwent right lobe hepatectomy, and the histological evaluation demonstrated granuloma with central caseous necrosis.

**Results:**

Seven months postoperatively, the patient remains fit and healthy.

**Conclusion:**

HTB is a rare entity with no specific symptoms, signs, and no laboratory nor imaging findings. It can be managed effectively if diagnosed in time or lead to death if left untreated.

## 1. Introduction

Tuberculosis (TB) is an infection which mainly affects the lungs. Extrapulmonary disease can occur at 15-20% of the patients, and less than 1% of these cases is located to the liver [1, 2]. Hepatic TB (HTB) can appear as a result of miliary tuberculosis or as a primary localized lesion [3]. Immunosuppressed patients are more likely to develop extrapulmonary disease or HTB [4]. There should be high clinical suspicion towards this entity, as the symptoms and the findings of HTB, such as abdominal pain or jaundice, are not specific, and the delay of diagnoses could lead to end-stage liver failure and death [5]. Last but not least, the diagnosis of HTB is based on histopathological and microbiological findings from the liver biopsy [6]. Herein, we repost a case of an immunocompetent female with primary HTB without any other sites of infection.

## 2. Case Presentation

A 50-year-old female patient referred to our hospital with a history of mild abdominal pain, located in the epigastrium and in the right upper quadrant, lasting for more than a month. In addition, she complained about weight loss (4 kg during last month), loss of appetite, and low-grade fever. At the time of administration, the patient weighted at 65 kg with a height of 162 cm (BMI: 24.77 kg/m^2^). There were no night sweats, cough, vomiting, nor dysuria. She suffered from hypertension, diabetes mellitus type 2, dyslipidemia, and seizures for which she was treated with the appropriate medication.

During the physical examination, the patient was presented with a blood pressure of 147/93 mmHg, pulse rate of 78/min, temperature of 37.4°C, and 17 breaths/min. She appeared pale. There was moderate tenderness of the right upper quadrant with hepatomegaly of three finger-breadths without splenomegaly, jaundice, nor ascites. The physical examination of the thorax did not reveal any abnormality, and no lymphadenopathy was detected.

Complete blood count revealed mild normocyte anemia (hemoglobin = 11.0 g/dl) and an erythrocyte sedimentation rate of 45 mm/h. Biochemical tests showed elevated alanine transaminase (355iU/L), aspartate aminotransferase (339iU/L), and alkaline phosphatase levels (302 iU/L) with normal renal function and electrolytes profile.

**Figure 1:**
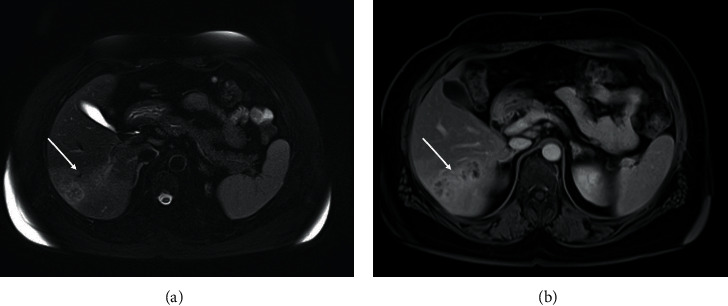
MRI scan which unveils the hepatic nodular lessions. (a) T1. (b) T2.

**Figure 2:**
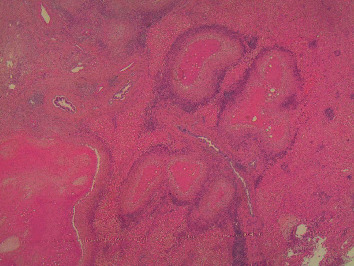
Liver section with multiple confluent, caseating granulomas (hematoxylin-eosin, original magnification ×20).

**Figure 3:**
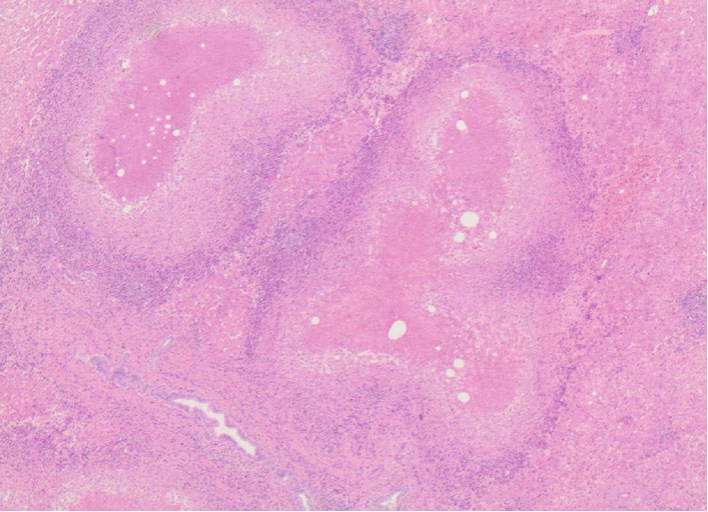
Multiple intrahepatic granulomas that coalesce to form nodules with central necrosis and a surrounding cuff of admixed inflammatory cells (hematoxylin-eosin, original magnification ×40).

The ultrasonography (US) of the abdomen demonstrated a mass in the right lobe of the liver characterized by mixed echogenity. An abdominal computed tomography (CT) followed, which demonstrated a hypodense mass in the right lobe of the liver next to a nonenhanced mass, with normal borders and a size of 1.8 cm, in the right adrenal gland consistent with a benign tumor. Neither chest X-ray nor CT revealed any other abnormal findings. The magnetic resonance imaging (MRI) of the abdomen showed hypointense and hyperintense multiple nodular lesions in T1-weighed imaging and T2 weighed-imaging, respectively, with abnormal borders with a greatest size of 2 cm (Figure 1). In addition, these lesions demonstrated enhancement after IV contrast administration. The initial differential diagnosis of these radiological findings included primary hepatocellular carcinoma or metastasic liver lesions. As a result, a core biopsy of liver mass with the assistance of ultrasound was conducted. It showed infiltration of predominant eosinoplils, lymphocytes, plasmatocytes, and some giant cells with epitheloid necrotic granulomas (Figure 2). No fungus or acid-fast bacilli were found in the specimen, and a parasite infection was suspected due to eosinophils presence, but it was unable to exclude malignancy or liver pseudotumor. A hepatectomy, including the VI segment of the right lobe of the liver, was performed, and the histopathological examination revealed multiple granulomas with central caseating necrosis. These findings are consistent with tuberculosis even though there were no acid-fast bacilli in Ziehl-Neelsen staining (Figure 3). A QuantiFERON-Tb test was conducted and turned out positive. Lastly, the patient was tested for human immunodeficiency virus (HIV) status, which was negative.

The antituberculous scheme, administrated to the patient, included isoniazid, rifampicin, pyrazinamide, and ethambutol for two months and isoniazid rifampicin for four months more. There were no serious adverse events observed, and all symptoms and laboratory findings were normal after completion of the treatment. Seven months postoperatively, the patient remains fit and healthy.

## 3. Discussion

Generally, HTB is extremely rare. Its frequency is slightly elevated in Asiatic populations. During the last decade, HTB arises in western countries due to immigration and due to the increasing number of patients with acquired immunodeficiency syndrome (AIDS) [7]. HTB is classified in three types which include the miliary TB derived from generalized infection, the primary hepatic miliary TB, and the rarest nodular lesion named tuberculoma [8, 9]. It is proposed that the entry point in the liver for the mycobacterium in the tuberculoma is the portal vein and in the miliary HTB is the hepatic artery [10].

The clinical presentation of this entity is not definitive. The most often symptoms and signs are low-grade fever, abdominal pain, tenderness of the right upper quadrant, and hepatomegaly [10]. Our patient did present all the above findings. The laboratory tests usually reveal an elevation of alkaline phosphatase with normal hepatic function, leukocytosis, and anemia [11]. The culture of the mycobacterium and the discovery of acid-fast bacilli are negative in the majority of the times in liver abscesses.

As far as the imaging methods are concerned, the US can demonstrate mostly hypoechoic lesions, while the typical CT finding is the heterogeneity of the lesions which vary from hypodense to hyperdense [12]. On T1-weighted imaging MRI, the lesions are hypointense, while on T2-weighted imaging, the lesions appear isointense and hyperintense with enhancement after contrast administration [13, 14]. In our case, there was a mixed echogenity in the ultrasound, hypodensity on CT, and hypointenisty and hyperintensity on T1- and T2-weighed imaging, respectively.

The diagnosis of HTB requires liver specimen acquired by laparotomy-, US-, or CT-guided biopsy. The diagnosis is confirmed by the presence of a caseating granuloma, or a noncaseating granuloma with positive culture for mycobacterium tuberculosis and/or acid-fast bacilli and improvement with anti-TB therapy [15]. Histologically, eosinophils, plasma cells, lymphohistiocytic cells, and langerhans-type giant cells are encountered in the granuloma [16]. The differential diagnosis includes mainly primary and metastatic liver malignancies.

The appropriate treatment for extrapulmonary TB is a 6 to 9 months scheme including administration of pyrazinamide, ethambutol, rifampin, and isoniazid for 2 months and afterwards, 4 to 7 months of rifampin and isoniazid [17].

Several articles and case reports in the literature have described interesting cases of isolated hepatic TB or in association with disseminated disease [18–23]. However, only Eshiwe et al. in 2019 [23] have clearly described hepatic involvement to such extent in an immunocompetent patient like in this case. More specifically, Abeysekera et al. [18], Kayar et al. [19], and Sharma et al. [20] described rare cases of isolated hepatic TB in immunocompetent patients. N'goran et al. [21] also described a case of military hepatic TB, and Çalışkan et al. [22] described a case of multiple hypoechoic lesions in a child, but none of them was a severe macronodular type involving the liver.

## 4. Conclusion

In conclusion, HTB is a rare entity with no specific symptoms, signs, laboratory nor imaging findings. Its hallmark is a central caseating necrotic granuloma with or without acid-fast bacilli. Thus, it is of paramount importance to suspect its presence in cases with diagnostic dilemmas and conduct liver biopsy were possible. HTB can be managed effectively if diagnosed in time and it can lead to death if left untreated.
